# Deciphering potential causative factors for undiagnosed Waardenburg syndrome through multi-data integration

**DOI:** 10.1186/s13023-024-03220-y

**Published:** 2024-06-06

**Authors:** Fengying Sun, Minmin Xiao, Dong Ji, Feng Zheng, Tieliu Shi

**Affiliations:** 1 Department of Clinical Laboratory, the Affiliated Wuhu Hospital of East China Normal University (The Second People’s Hospital of Wuhu City), Wuhu, 241000, China; 2 Department of Otolaryngology, Head and Neck Surgery, the Affiliated Wuhu Hospital of East China Normal University (The Second People’s Hospital of Wuhu City), Wuhu, 241000, China; 3 Wuhu Hospital and Health Science Center, East China Normal University, Shanghai, 200241, China; 4 Center for Bioinformatics and Computational Biology, the Institute of Biomedical Sciences and the School of Life Sciences, East China Normal University, Shanghai, 200241, China; 5 Beijing Advanced Innovation Center, for Big Data-Based Precision Medicine, Beihang University & Capital Medical University, Beijing, 100083, China

**Keywords:** Hereditary deafness, Waardenburg syndrome, New potential pathogenic genes, New potential disease-causing variants, Genotype, Phenotype

## Abstract

**Background:**

Waardenburg syndrome (WS) is a rare genetic disorder mainly characterized by hearing loss and pigmentary abnormalities. Currently, seven causative genes have been identified for WS, but clinical genetic testing results show that 38.9% of WS patients remain molecularly unexplained. In this study, we performed multi-data integration analysis through protein-protein interaction and phenotype-similarity to comprehensively decipher the potential causative factors of undiagnosed WS. In addition, we explored the association between genotypes and phenotypes in WS with the manually collected 443 cases from published literature.

**Results:**

We predicted two possible WS pathogenic genes (*KIT*, *CHD7*) through multi-data integration analysis, which were further supported by gene expression profiles in single cells and phenotypes in gene knockout mouse. We also predicted twenty, seven, and five potential WS pathogenic variations in gene *PAX3*, *MITF*, and *SO*X10, respectively. Genotype-phenotype association analysis showed that white forelock and telecanthus were dominantly present in patients with *PAX3* variants; skin freckles and premature graying of hair were more frequently observed in cases with *MITF* variants; while aganglionic megacolon and constipation occurred more often in those with *SOX10* variants. Patients with variations of *PAX3* and *MITF* were more likely to have synophrys and broad nasal root. Iris pigmentary abnormality was more common in patients with variations of *PAX3* and *SOX10*. Moreover, we found that patients with variants of *SOX10* had a higher risk of suffering from auditory system diseases and nervous system diseases, which were closely associated with the high expression abundance of *SOX10* in ear tissues and brain tissues.

**Conclusions:**

Our study provides new insights into the potential causative factors of WS and an alternative way to explore clinically undiagnosed cases, which will promote clinical diagnosis and genetic counseling. However, the two potential disease-causing genes (*KIT*, *CHD7*) and 32 potential pathogenic variants (*PAX3*: 20, *MITF*: 7, *SOX10*: 5) predicted by multi-data integration in this study are all computational predictions and need to be further verified through experiments in follow-up research.

**Supplementary Information:**

The online version contains supplementary material available at 10.1186/s13023-024-03220-y.

## Background

Waardenburg syndrome (WS), also known as an auditory-pigmentary syndrome, is the most common cause of autosomal-dominant syndromic deafness with an estimated prevalence of 1:42000 in the general population but is present in 0.9-2.8% of the deaf population and in 2-5% of patients with congenital deafness [1, 2]. Based on different concomitant phenotypes, WS is subdivided into four subtypes (WS1 to WS4) [3]. The current study indicates that WS1 (OMIM:193,500) and WS2 (OMIM:193,510) are more common than WS3 (OMIM:148,820) and WS4 (OMIM:277,580) in the clinic [4–7]. WS2 is highly phenotypically similar to WS1, but WS2 lacks dystopia canthorum which is specific for WS1 with a penetrance of 97% [8]. WS3, also called Klein-Waardenburg syndrome, is the severe presentation of WS1. WS3 is characterized by the presence of upper limb abnormalities (cutaneous finger syndactyly, camptodactyly of finger, and joint contracture of the hand) in addition to the common phenotypes of WS1 [9, 10]. WS4 (Waardenburg-Shah syndrome) is characterized by WS2 features as well as Hirschsprung’s disease (OMIM:142,623), a disorder caused by the congenital deficiency of neural crest from enteric ganglia that causes severe blockage of the large intestine [5, 11].

To date, variations in seven genes, namely paired box 3 (*PAX3*) [7], melanocyte inducing transcription factor (*MITF*) [7], snail family transcriptional repressor 2 (*SNAI2*) [12], SRY-box transcription factor 10 (*SOX10*) [7], endothelin 3 (*EDN3*) [5], endothelin receptor type B (*EDNRB*) [5], *KIT* ligand (*KITLG*) [13], have been confirmed to be related to WS. The molecular aetiologies overlap between the different subtypes. *PAX3* is related to WS1, WS2, and WS3 [5, 7, 9], *MITF* and *SOX10* are associated with WS1, WS2, and WS4 [5, 7, 11, 14, 15], *EDN3* is linked to WS4 [16], mutations in *EDNRB* gene cause WS1 and WS4 [5, 17], and some WS2 cases result from the mutations in the *SNAI2* and *KITLG* genes [12, 13].

In large cohort or family studies, previous researchers mainly used targeted known WS pathogenic genes or deafness-related genes capture and next-generation sequencing for simultaneous single-nucleotide variants and copy number variation detection in WS [5, 7, 18, 19]. However, targeted sequencing has a low-resolution rate compared to whole exome sequencing or whole genome sequencing, which could miss the other disease-related genes or variants, leaving some WS cases to remain unexplained at the molecular level. For example, Wang et al. have reported that 38.9% (35/90) of probands in their research have no disease-causing variants detected [7]. Similarly, genetic analyses in a study by Li et al. revealed that 14.8% (4/27) of WS1 and 26.3% (15/57) of WS2 cases remained molecularly unexplained [5]. These results suggest that new potential WS-related genes may be involved in the disease. There is also the possibility that some variants within the known genes are not detected. Thus, novel potential pathogenic genes and variants remain to be discovered.

As a heterogeneous disorder, WS patients have different clinical manifestations because of mutations of different genes. A genotype-phenotype association study by Wang et al. [7] has revealed that brown freckles on the skin occur significantly more often in cases with *MITF* variants but not in those with *SOX10* or *PAX3* variants. White forelock and patchy depigmented skin occur frequently in probands with *PAX3* variants but are absent in those with *SOX10* or *MITF* variants. These results indicate that these unique phenotypes could be used to distinguish the genotype of WS. However, due to the small sample size, there are some biases between the results of previous association studies. For instance, Zhang et al. studied the genotype-phenotype correlations and discovered that synophrys was only present in WS1 patients with *PAX3* mutations [20]. However, Haddad et al. also reported an association of *MITF* mutations with synophrys symptoms in two WS2 patients [17].

A study by Li et al. indicates that pigmentation spots, congenital ptosis, yellow hair, amblyopia, and narrow palpebral fissures are rare and unique symptoms in WS patients from China [5]. However, their study has certain limitations as all the patients they included were from China. In addition, Suzuki et al. and Rauschendorf et al. have reported that probands from other countries also have pigmentation spots and congenital ptosis manifestations [15, 21]. Thus, in order to provide more accurate genotype-phenotype associations and avoid research bias caused by small sample size or regional clinical cases, comprehensive association analysis is required in large WS-cohort with different ethnic backgrounds.

In this research, we first predicted four new possible putative WS pathogenic genes (*SIN3A*, *EP300*, *CHD7*, and *KIT*) by multi-data integration. Among them, *KIT* and *CHD7* were more associated with WS than *SIN3A* and *EP300*. Next, we predicted several possible putative disease-causing variants in *PAX3*, *MITF*, and *SOX10* by ANNOVAR annotation. Subsequently, we collected genotypic and phenotypic information on 443 WS cases from published literature and then analyzed the genotype-phenotype correlations based on the clinical information of 423 patients with *PAX3*, *MITF*, or *SOX10* variants. Considering the high degree of genotypic and phenotypic heterogeneity of WS and the limited size of the patients available, our comprehensive analysis could provide more precise description of the variants responsible for WS to clarify the molecular factors of clinical characteristics of WS and provide better genetic counseling for WS patients and their families.

## Methods

### Patient data information

We searched PubMed to collect reported cases. The search results were filtered solely for WS. Detailed description of phenotypic features and variant information of patients with WS were the two important collection criteria. After manual retrieving and detailed reading of the literature, the information of 443 WS patients with different ethnic backgrounds from 84 published studies was manually collected (WS1: 125 (28.22%), WS2: 255 (57.56%), WS3: 5 (1.13%), WS4: 31 (7%), unclassified: 27 (6.09%)), including country, gender, age, genotypes, phenotypes, and variant types. The 443 WS patients were from 288 unrelated families, of which 223 cases from 65 families were familial and the other 220 cases were sporadic. Since the variants in *PAX3*, *MITF* and *SOX10* concern a majority of WS cases and the variants in *EDNRB*, *EDN3*, *SNAI2* and *KITLG* are responsible for the minority of patients (total 443, *PAX3*: 138 (31.15%), *MITF*: 161 (36.34%), *SOX10*: 124 (27.99%), *EDNRB*: 13 (2.93%), *EDN3*: 4 (0.90%), *SNAI2*: 2 (0.45%) and *KITLG*: 1 (0.23%)), our research was only focused on those patients with variants in *PAX3*, *MITF* and *SOX10* (total 423) to analyze the genotype-phenotype correlations. No conclusion currently has been made whether there is a gender difference in the prevalence of phenotypes shared by female and male patients with different gene variants. Of the 423 patients with *PAX3*, *MITF*, or *SOX10* variants, 310 patients with gender records (99 patients with *PAX3* variants (male: 46, female: 53), 86 patients with *SOX10* variants (male: 47, female: 39), 125 patients with *MITF* variants (male: 66, female: 59)) were used to analyze the difference of phenotypic distribution in different genders. The clinical information of 443 WS patients is shown in Additional file 1.

### Public database resources

The large number of protein-protein interactions (PPI) accumulated in the past decade play an important role in disease mechanism study and have been frequently used to predict novel candidate proteins since they offer functional information in a network environment [22, 23]. In this study, we used PPI databases (BioGRID, HitPredict, InWeb_InBioMap) to predict possible putative WS pathogenic genes.

HPO, a Human Phenotype Ontology database, provides a standardized vocabulary of phenotypic abnormalities encountered in human disease. HPO currently contains over 13,000 terms, over 156,000 annotations to hereditary diseases, and over 4,700 genes with corresponding phenotypes [24]. We standardized the phenotypic description for collected 443 patients based on the HPO database. In addition, we download the phenotypic data of 4,707 genes from the HPO database.

MORL scRNA-Seq database contains the single-cell RNA-Seq data of inner hair cells (IHCs) (*n* = 42), outer hair cells (OHCs) (*n* = 127), and Deiters’ cells (DCs) (*n* = 39) from a total of 70 c3HeB/FeJ mice. The database can be used to analyze rare and difficult-to-isolate cell types from the mature organ of Corti and identify cell type-defining genes [25]. We queried the gene expression abundance of those predicted candidate pathogenic genes from the database. Studying gene expression abundance at the single-cell level of the cochlea can improve our understanding of the biology of hearing and deafness.

Mouse Genome Informatics (MGI), an international resource for information on the laboratory mouse, contains these interacting databases: Mouse Genome Database, Gene Expression Database, International Mouse Strain Resource, and the Mouse Tumor Biology Database. Data include over 78,000 expression assays, 300,000 GO annotations, and 300,000 mammalian phenotype annotations [26].

The Human Protein Atlas database wide analysis of the protein expression covering more than 90% of the putative protein-coding genes in all major tissues and organs (*n* = 44), and analysis of the RNA-sequencing data for 32 of the tissues, providing the possibility to explore gene expression profiles in tissues [27].

### New candidate pathogenic gene prediction

**Fig. 1:**
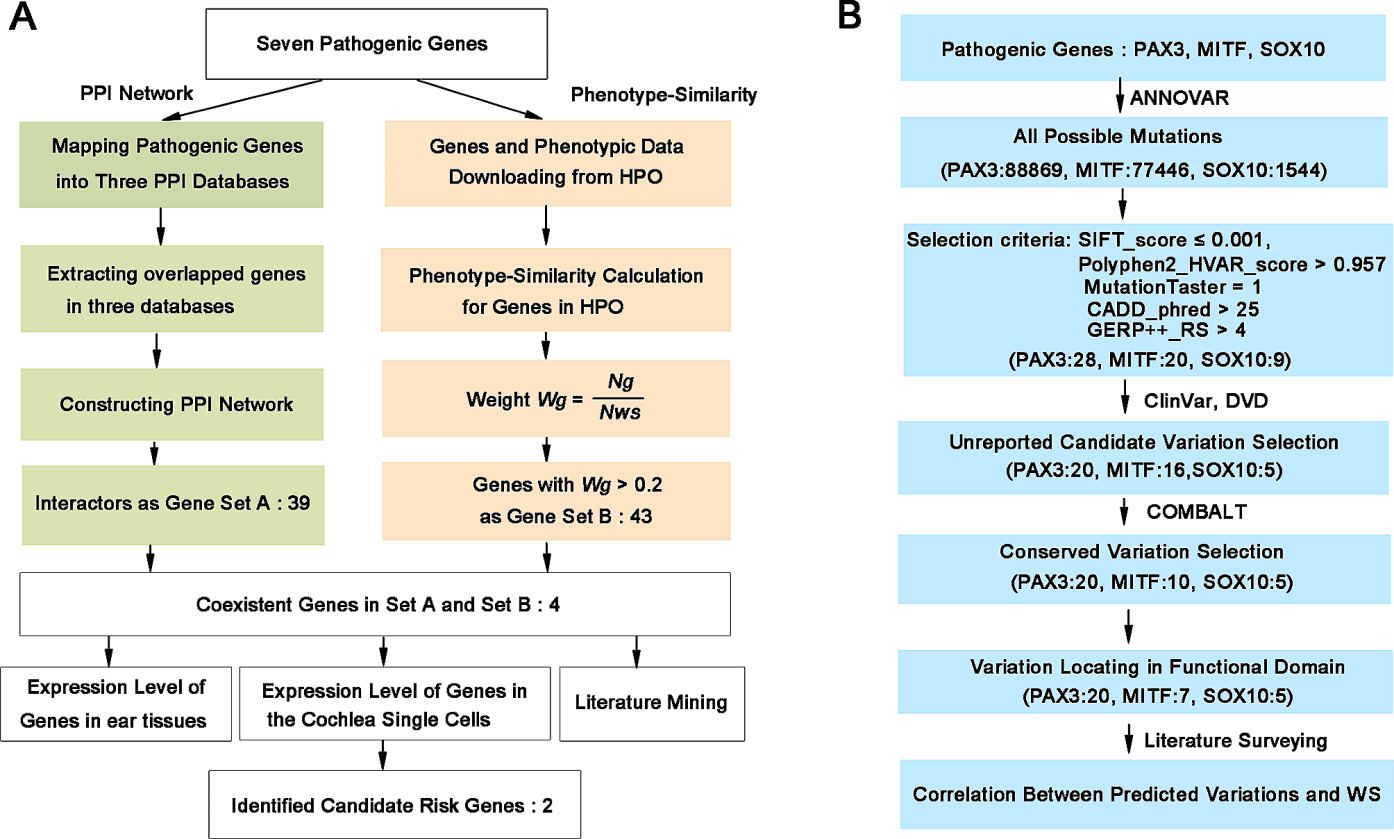
Detailed flow charts for the analysis process. **(A)** A detailed flow chart for WS-related pathogenic gene prediction. **(B)** A detailed flow chart for WS-related disease-causing variations prediction

We performed a systematical analysis through multi-data integration to discover novel potential WS pathogenic genes. The whole process is displayed in Fig. 1A. Firstly, we mapped 7 experimentally validated WS causative genes (*PAX3*, *MITF*, *SOX10*, *SNAI2*, *EDNRB*, *EDN3*, *KITLG*) into BioGRID, InWeb_InBioMap and HitPredict databases respectively. Then we selected their directly interacting protein pairs (the predicted gene set A) shared in these three databases to construct a PPI network consisting of those pathogenic genes and their interaction pairs with Cytoscape software.

Next, we used the phenotype-similarity approach to select those candidate pathogenic genes whose weight *W*_*g*_ > 0.2 as predicted gene set B. The phenotype-similarity method was described in detail in previous literature [28]. Briefly, the core idea of the phenotype-similarity method is that the more overlap between the phenotypes caused by gene variations and phenotypes linked to a disease, the stronger the relationship between the gene and the disease. We first obtained a unified set of phenotypes related to WS based on 7 pathogenic genes from the HPO database, defined as *P*_*ws*_. In addition, we defined *P*_*g*_ as a corresponding phenotype set for a gene in the HPO database. We then extracted the overlap phenotypes between the phenotype set *P*_*g*_ of each gene in HPO and the phenotypes set *P*_*ws*_ respectively and calculated the weight *W*_*g*_ of every gene. The Weight *W*_*g*_ was defined as Weight *W*_*g*_ = *N*_*g*_/*N*_*ws*_. *N*_ws_ denotes the number of phenotypes in set *P*_*ws*_. *N*_*g*_ denotes the number of overlapped phenotypes in set *P*_*ws*_ and *P*_*g*_. We selected those genes whose weight *W*_*g*_ > 0.2 as predicted pathogenic gene set B. The *W*_*g*_ cutoff was set to > 0.2 in our study. There are two main reasons. Firstly, *W*_*g*_=*N*_*g*_/*N*_*ws*_ (*N*_*ws*_ = 70), it is equivalent to selecting genes with overlapping phenotypes > 14 as candidate gene set B. Secondly, it is equivalent to selecting the top 50 genes from 4,707 downloaded genes. Finally, we extracted those overlapped genes between the gene set A and the gene set B, defined as the preliminary candidate genes.

Subsequently, we looked at the phenotypes of these genes with mutations in mouse based on the MGI database and selected those genes whose mutations cause hearing impairment in mouse as high-priority candidate pathogenic genes. We then retrieved the expression level of these high-priority candidate pathogenic genes in different mouse ear tissues based on the MGI database [26]. In addition, we mapped these candidate pathogenic genes into the MORL scRNA-Seq database and retrieved the expression abundance of these genes in the single cell level of the cochlea [25]. Finally, we conduct extensive literature mining in PubMed to further confirm those new potential pathogenic genes of WS discovered by integrating multiple databases.

### New candidate disease-causing variations identification

The previous report indicated that 38.9% of WS cases remained molecularly unexplained. One possible reason is that some variants in known disease-causing genes have not discovered. Considering *PAX3*, *MITF*, and *SOX10* are the main causative genes of WS, we only predicted novel WS-related pathogenic variants in *PAX3*, *MITF*, and *SOX10* genes. We first used the “-downdb -webfrom annovar” command in the Linux operating system to downloaded variant information of all genes from gnomad_genome, exac03, and avsnp147 databases. Then we used the “perl table_annovar.pl” command to annotate variants. Finally, annotation information of *PAX3*, *MITF*, and *SOX10* gene variants were extracted.

ANNOVAR tool offers functional annotation of single nucleotide variants and insertions/deletions with over 10 different software, including SIFT, PolyPhen2, MutationTaster, CADD, GERP++, and so on. SIFT is a program that uses sequence homology to predict whether an amino acid substitution in a protein affects protein function and may contribute to phenotypic differences. SIFT predicts scores less than 0.05 as deleterious [29]. PolyPhen-2 is a tool for the prediction of the possible impact of amino acid substitutions on the stability and function of human proteins based on several sequences, phylogenetic, and structural features characterizing the substitution [30]. MutationTaster predicts the disease-causing potential of DNA sequence alterations by analyzing the evolutionary conservation, loss of protein features, splice-site changes, and changes that might affect the amount of mRNA [31]. CADD is a widely used measure of variant deleteriousness, which ranks the pathogenicity of genetic variants based on diverse genomic features, evolutionary constraints, epigenetic measurements, gene model annotations, and functional predictions [32]. GERP + + regards those nucleotide substitution deficits as “rejected substitutions”, which can measure the intensity of evolutionary constraint on each aligned position [33]. The pathogenicity of each variation on *PAX3*, *MITF*, and *SOX10* genes is predicted by the above five software with defined cutoff values (SIFT_score ≤ 0.001, Polyphen2_HVAR_score > 0.957, MutationTaster = 1, CADD_phred > 25, GERP++_RS > 4).

To further analyze the pathogenic ability of those predicted variants, we used ClinVar (https://www.ncbi.nlm.nih.gov/clinvar/) and Deafness Variation Database (DVD) [34] to check if those predicted variants have been reported to be pathogenic to WS. We then performed multiple-sequence alignment using COMBALT software and identified the conservation of those predicted variants [35]. Finally, we searched the functional domain information of each predicted variant by text mining. The whole process is displayed in Fig. 1B.

### Statistical analysis

All the calculated process was performed using SPSS software 19.0. Two-sided Pearson’s Chi-square tests or two-sided Fisher’s exact tests were used to compare the prevalence of each phenotype among WS patients with different gene mutations. The degree of correlation between any two phenotypes was calculated with the Phi correlation coefficient (ϕ). The range of the ϕ coefficient was in the range from − 1 to 1, suggesting that the closer the value is to the poles, the higher the correlation. The two-sided Pearson’s Chi-square tests are used to calculate the correlation *P*-value between any two phenotypes. *P* < 0.05 is considered statistically significant.

## Results

### New candidate pathogenic gene identification

**Fig. 2:**
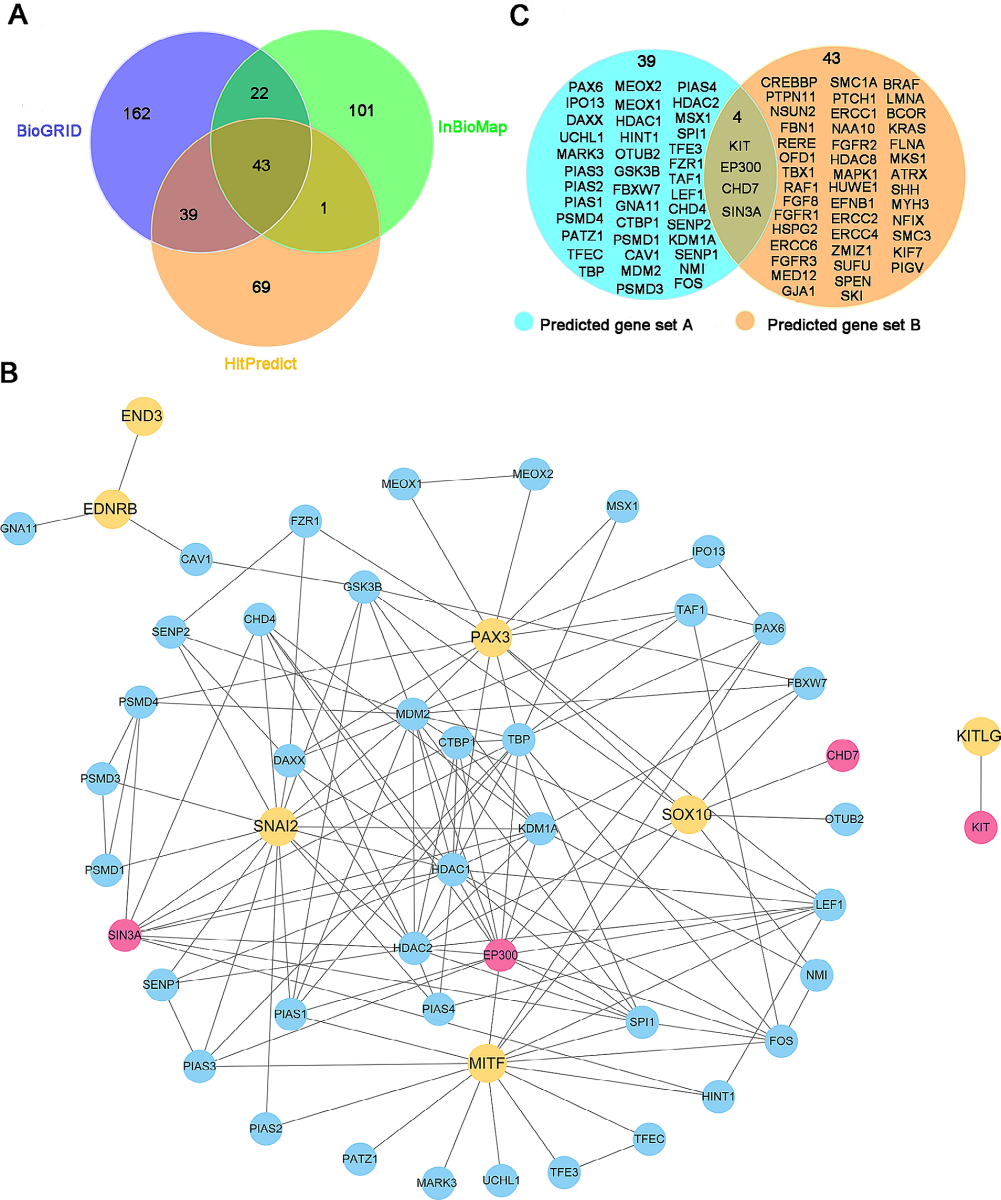
Prediction of new candidate pathogenic genes for WS by PPI and phenotype-similarity approach. **(A)**Venn diagram of candidate genes predicted through three PPI databases. The numbers in purple, green and orange circles represent the numbers of interacting proteins predicted by the BioGRID, InWeb_InBioMap and HitPredict databases respectively. **(B)** PPI network of WS causative genes and candidate genes. 141 interactions between 7 WS causative genes and 43 interacted genes. Nodes in yellow denote the pathogenic genes. Nodes in blue and red denote the interacted genes. Nodes in red denote the new candidate genes. **(C)** Venn diagram of PPI network and phenotype-similarity network. The genes in the blue circle denote candidate genes predicted by three PPI databases. The genes in the orange circle represent candidate genes predicted by the phenotype-similarity method (the numbers of phenotypes shared with pathogenic genes ≥ 15). 4 genes (*SIN3A*, *EP300*, *CHD7*, and *KIT*) both exist in PPI network and phenotype-similarity networks, defined as the preliminary candidate pathogenic genes

**Fig. 3:**
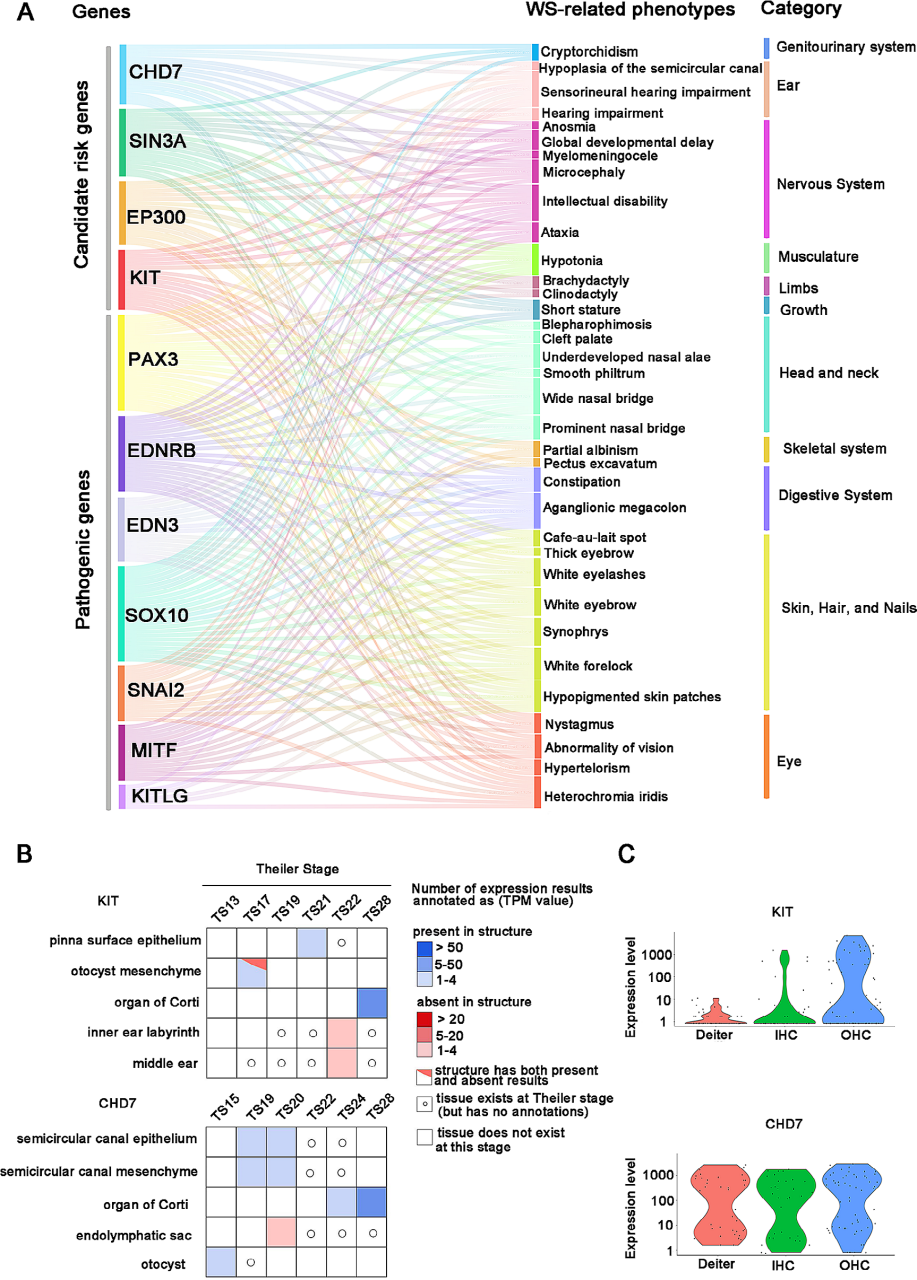
The WS-related phenotypes shared by causative genes and candidate genes and expression abundance of candidate genes in ear tissues. **(A)** A Sankey diagram displays the WS-related phenotypes shared by causative genes and candidate genes. *SIN3A*, *EP300*, *CHD7*, and *KIT* share 17, 16, 15, and 15 phenotypes with causative genes, respectively. **(B)** Tissue × stage expression matrix of *KIT* and *CHD7* genes in ear tissues. Data is from the MGI database [26]. **(C)** Violin plot showing gene expression levels of *KIT* and *CHD7* in DCs, IHCs, and OHCs of c3HeB/FeJ mice. Data is from the MORL scRNA-Seq database [25]

To predict new pathogenic genes associated with WS, we conducted a network-based integrative analysis based on the principle that interacting proteins have function association between them [28]. First, based on the PPI data, we respectively obtained 266, 167 and 152 genes that directly interact with causative genes through mapping 7 known disease causing genes (*PAX3, MITF, SOX10, SNAI2, EDNRB, EDN3, KITLG*) into BioGRID, InWeb_InBioMap and HitPredict databases, respectively (Fig. 2A). To improve the prediction accuracy, we extracted 43 proteins that overlapped in the three databases as candidate gene set A and constructed a PPI network (Fig. 2B). Next, we downloaded a total of 4,707 genes and their corresponding phenotypes from the HPO database. We obtained a unified phenotypic set *P*_*ws*_ containing 70 phenotypes associated with WS by mapping seven pathogenic genes into HPO. Through comparing the phenotypic set *P*_*g*_ of each gene in HPO with *P*_*ws*_ and filtering out those genes with weight *W*_*g*_ < 0.2, a gene set B of 47 genes was obtained (Fig. 2C). Subsequently, we extracted those overlapping genes (*SIN3A*, *EP300*, *CHD7*, and *KIT*) between gene set A and gene set B as potential WS pathogenic genes. Sankey diagram shows that the 4 candidate pathogenic genes have a total of 35 WS-related phenotypes. *SIN3A*, *EP300*, *CHD7*, and *KIT* genes share 17, 16,15, and 15 phenotypes with causative genes, respectively (Fig. 3A).

Among the abnormal clinical manifestations of WS, hearing loss is the primary symptom affecting WS patients’ health and quality of life [36]. To further confirm the four potential WS pathogenic genes, we retrieved the phenotypes of these four candidate genes in mouse from the MGI database and found that both *KIT* and *CHD7* mutations could cause hearing loss in mouse (Additional file 2, Additional file 3). Next, we retrieved the expression abundance of *KIT* and *CHD7* in different ear tissues based on the MGI database. The results show that both *KIT* and *CHD7* are mainly highly expressed in the organ of Corti in the cochlea (Fig. 3B). The organ of Corti acts as an auditory receptor, and its damage leads to varying degrees of sensorineural hearing loss. The sensory epithelium of the organ of Corti is made up of HCs and SCs. HCs are sensory cells, and they are key components in sound perception. We then further retrieved the expression level of *KIT* and *CHD7* in cochlea single cells based on the MORL scRNA-Seq database. The results show that *KIT* is mainly highly expressed in OHCs. *CHD7* is highly expressed in IHCs, OHCs, and DCs (Fig. 3C). Taken together, the above results indicate that *KIT* and *CHD7* could be high-priority candidate pathogenic genes contributing to WS.

### The identification of disease-causing variations in WS-related genes

We obtained 88,869 possible variations of *PAX3*, 77,446 possible variations of *MITF*, and 1,544 possible variations of *SOX10* by ANNOVAR annotation. After filtering out variations based on the cutoff value (SIFT_score ≤ 0.001, Polyphen2_HVAR_score > 0.957, MutationTaster = 1, CADD_phred > 25, GERP++_RS > 4), 28 *PAX3* variations, 20 *MITF* variations, and 9 *SOX10* variations were obtained and kept as candidate disease-causing mutations (Additional file 4). Strikingly, 16 of the 57 variations have been reported to be pathogenic to WS in ClinVar and DVD (Additional file 4). The remaining 20 candidate variations in *PAX3*, 16 candidate variations in *MITF*, and 5 candidate variations in *SOX10* may be considered as potential disease-causing variants.

Next, the multiple sequence alignment of *PAX3*/*MITF*/*SOX10* homologous genes among 7 species (*Homo sapiens*, *Macaca mulatta*, *Pan troglodytes*, *Mus musculus*, *Equus caballus*, *Sus scrofa*, *Canis lupus familiaris*) with COMBALT showed that 20 variations in *PAX3*, 10 variations in *MITF* and 5 variations in *SOX10* located in highly conserved regions among those species (Additional file 4). Subsequently, we checked the position of those candidate disease-causing variations in protein structure and found 20 variations (p.Gln470Leu, p.Tyr458His, p.Gln431Glu, p.Val114Met, p.Tyr366Asn, p.Pro244Leu, p.Gly42Cys, p.Gly34Cys, p.Gly48Ala, p.Gly34Arg, p.Arg156Cys, p.Ser152Asn, p.Pro382His, p.Asn47Lys, p.Gly417Arg, p.Trp131Ser, p.Pro333Leu, p.Asp144Tyr, p.Arg220Cys, p.Arg156His) in *PAX3*, 7 variations (p.Arg263Gly, p.Arg279Leu, p.Pro232Arg, p.Arg279Trp, p.Arg263Cys, p.Arg263Leu, p.Glu287Lys) in *MITF* and 5 variations (p.Thr437Met, p.Arg465Leu, p.Thr461Met, p.Pro245Arg, p.Pro302Leu) in *SOX10* located in domain regions (Additional file 4, Fig. 4), which may influence the protein structure, interaction and function. Thus, we proposed that these 32 candidate mutations may be closely associated with WS disease.

**Fig. 4:**
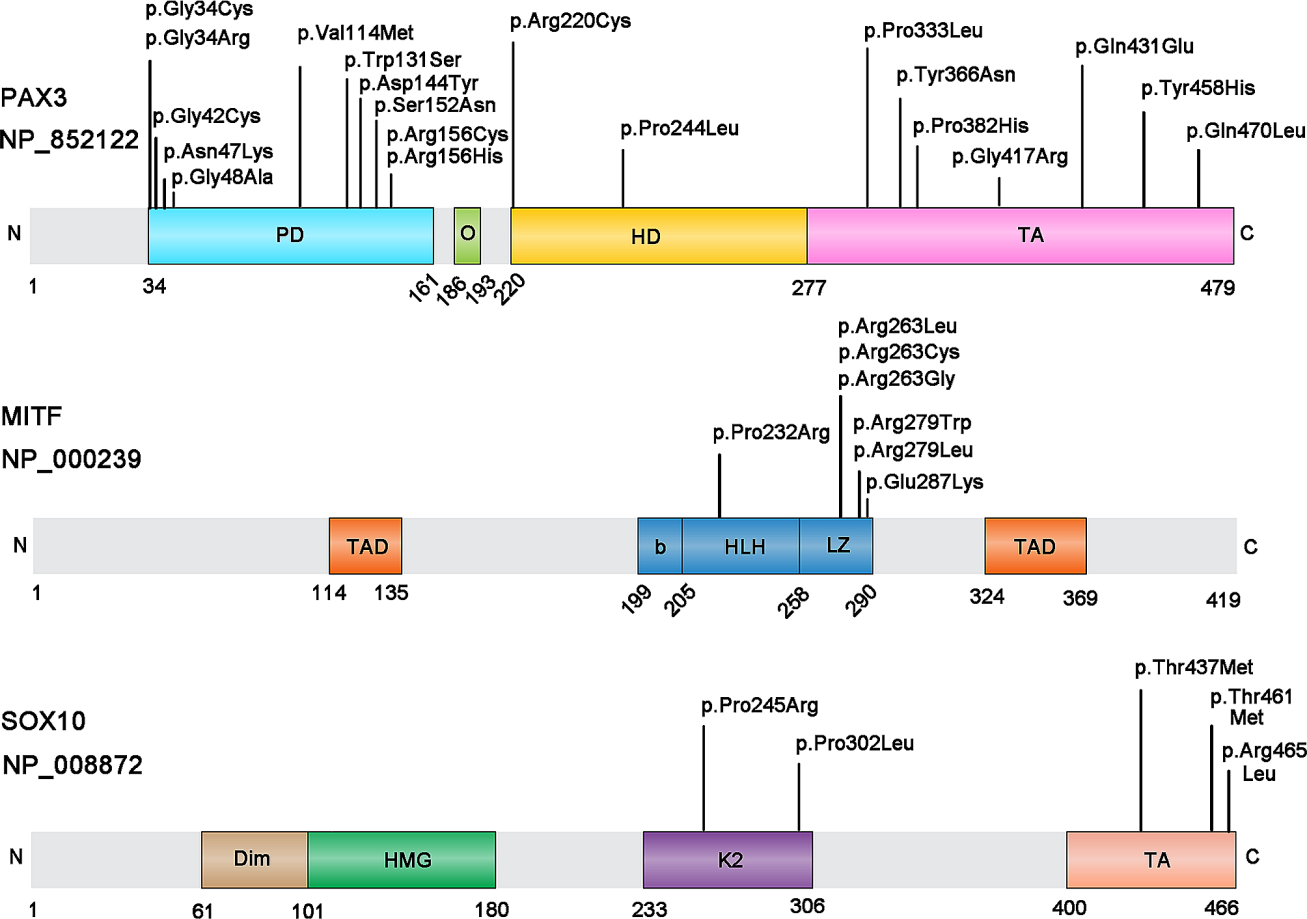
Position of candidate disease-causing variants in protein domains. **(A)** Structure of human *PAX3* protein with functional domains. PD, paired domain; O, octapeptide motif; HD, homeodomain; TA, transactivation domain. **(B)** Structure of human *MITF* protein with functional domains. TAD, transactivation domain; b-HLH-Zip, basic helix-loop-helix leucine zipper. **(C)** Structure of human *SOX10* protein with functional domains. Dim, dimerization domain; HMG, high-mobility group; K2, K2 domain; TA, transactivation domain

### The distribution of genes and variation types

**Fig. 5:**
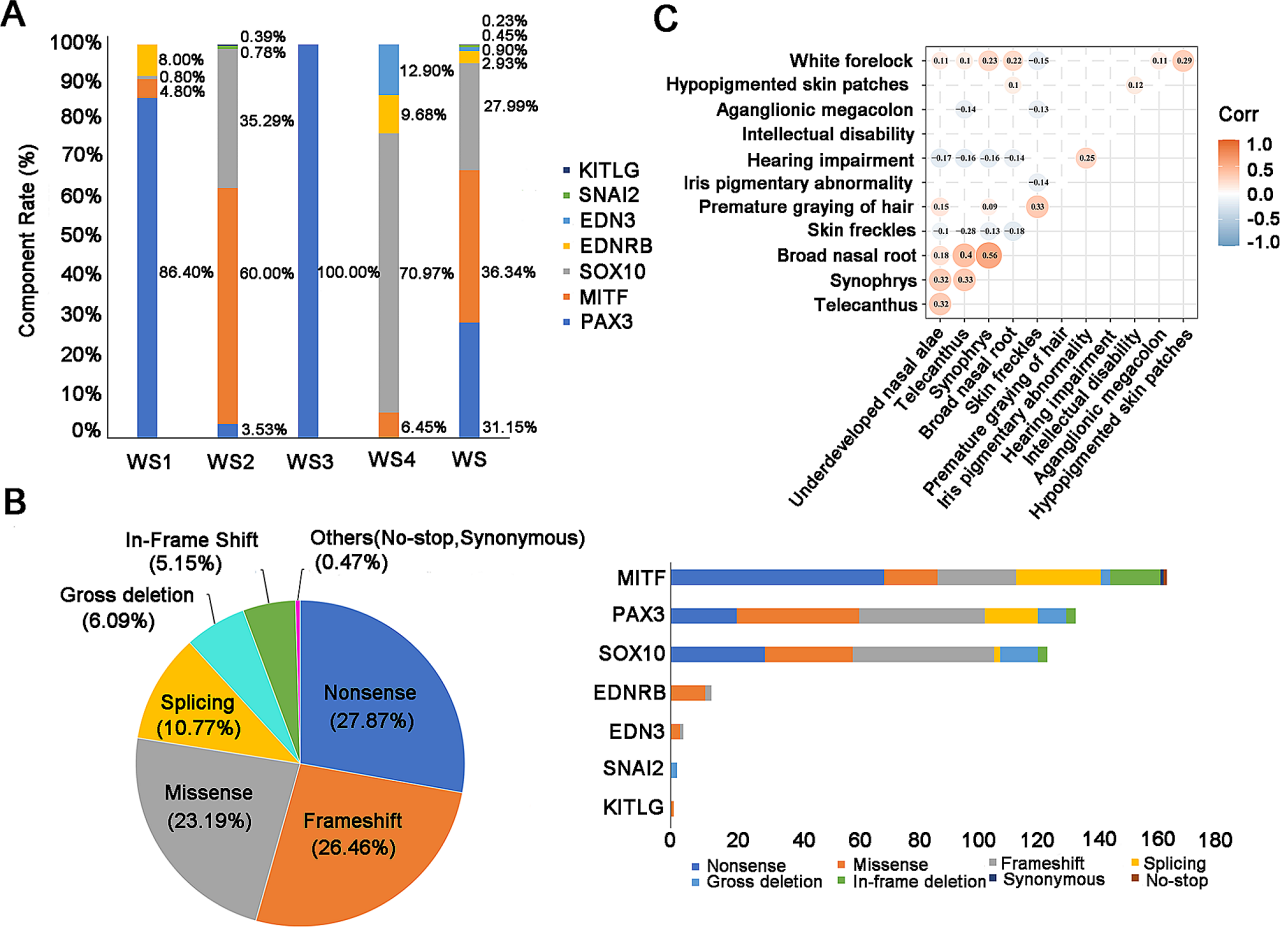
The distribution of genes and variant types. **(A)** The gene distribution of four WS subtypes. **(B)** The distribution of mutation types in WS (the left figure); the distribution of mutation types of 7 pathogenic genes in WS (the right figure). **(C)** A heatmap was used to show the correlation between any two phenotypes in WS. The circle shown in the figure suggests that the correlation it represents has passed the statistical test. The value on the circle is used to evaluate the degree of relationship. Red circles indicate that the two phenotypes are positively correlated. Blue circles indicate that the two phenotypes are negatively correlated

Information on 443 WS cases was collected from 84 published literature. The most common genetic causes were *MITF* variants (36.34%), *PAX3* variants (31.15%), and *SOX10* variants (27.99%), which accounted for 95.48% of molecular diagnosed WS patients (Fig. 5A). *PAX3* variants were the most frequent genetic causes of WS1 and WS3 patients, which accounted for 86.40% and 100.00% respectively. The most common genetic factors of WS2 and WS4 were *MITF* variants (60.00%) and *SOX10* variants (70.97%) respectively (Fig. 5A).

Nonsense (27.87%), frameshift (26.46%), missense (23.19%), and splicing (10.77%) mutations occupied 88.29% of total causative variants. Relatively frequent variants were also observed, including gross deletion (6.09%) and in-frame deletion (5.15%) (Fig. 5B) (the left figure). Nonsense mutations were the majority of variants in *MITF* gene. Missense and frameshift mutations were more common in *PAX3*, *SOX10*, *EDNRB*, *EDN3*, and *KITLG* (Fig. 5B) (the right figure).

### The position distribution of known disease-causing variants related to WS

The gene structure diagram displayed the common disease-causing variants associated with WS in 7 pathogenic genes (Additional file 5). Results showed that the majority of variants (95.98%) occurred in exon regions in seven pathogenic genes. Thus, screening of whole exons can be prioritized in genetic testing. Currently, whole-exome sequencing has been widely used in the diagnosis of genetic diseases [37, 38]. However, since the seven genes also have a few variants that occur in intron regions, the intron-exon boundaries should also be inspected during genetic testing. Moreover, as displayed in Fig. 5B (the right figure), gross deletions account for 1.90%, 6.98%, and 10.00% of *MITF*, *PAX3*, and *SOX10* variants, respectively. Thus, when WS cases with no variants were detected in exons and introns, structure variations should be considered. In addition, approximately 39% of WS, 14.8% of WS1, 26.3% of WS2, and 15–35% of WS4 patients remain unknown for the pathogenic genes [5–7]. Considering the rapid development of sequencing technology with significantly decreased in cost and time [39, 40], whole genome sequencing can be a prioritized approach when the conventional clinical genetic testing methods fail to detect the disease-causing variants for WS.

### Genotype-phenotype correlation analysis

*PAX3*, *MITF*, and *SOX10* are the main pathogenic genes of WS patients, which accounted for 95.48% of our collected 443 cases from published literature (Fig. 5A). To avoid analysis bias resulted from the insufficient reported cases, the cases for the other known WS-causing genes (*EDNRB*, *EDN3*, *SNAI2*, and *KITLG*) were excluded for subsequent analysis. Additional file 6 summarizes the frequencies of the 13 most common phenotypes among our collected patients with variants in *PAX3* (*n* = 138), *MITF* (*n* = 161), and *SOX10* (*n* = 124). We conducted association analysis to investigate whether two phenotypes were prone to coexist among these common phenotypes. Results revealed that the occurrence of telecanthus and synophrys was linked to the broad nasal root (corr = 0.4/0.56) (Fig. 5C). However, some phenotypic pairs passed the statistical test with relative low correlation coefficient, more clinical cases are needed to confirm the association.

**Fig. 6:**
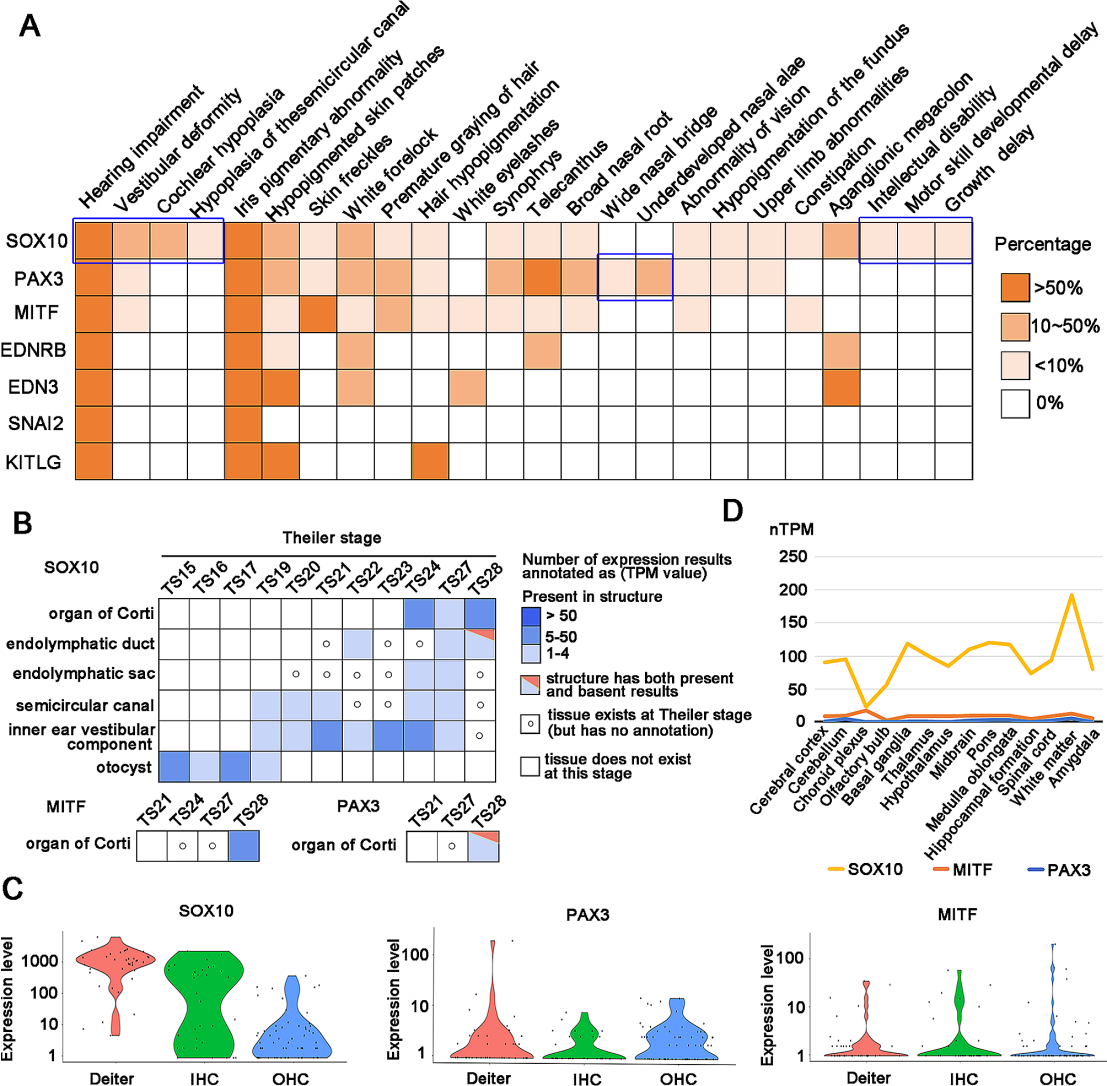
The common phenotypes of WS and the expression abundance of genes in ear tissues and brain tissues. **(A)** The heatmap showed the percentage of 24 common phenotypes in WS. **(B)** Tissue × stage expression matrix of *SOX10*, *MITF*, and *PAX3* genes in ear tissues. MGI database only annotates the expression of *MITF* and *PAX3* in the organ of Corti. Compared with *PAX3* and *MITF*, *SOX10* was widely and highly expressed in the organ of Corti, semicircular canal, inner ear vestibular component and otocyst. Data is from the MGI database [26]. **(C)** Violin plot showing gene expression levels of *SOX10*, *MITF*, and *PAX3* in DCs, IHCs, and OHCs of c3HeB/FeJ mice. The expression level of *SOX10* is higher than *MITF* and *PAX3* in cochlear single cells. Data is from the MORL scRNA-Seq database [25]. **(D)** Line chart showing the expression level of *SOX10*, *PAX3*, and *MITF* in different brain tissues. *SOX10* has a higher expression level than *MITF* and *PAX3* in different brain tissues. The yellow, orange and blue lines denote pathogenic genes *SOX10*, *MITF*, and *PAX3*, respectively. Data is from the Human Protein Atlas database [27]

De novo variants were more common in patients with *SOX10* variants (61.70%) than patients with *MITF* or *PAX3* variants (*p* = 5.2013E-11 for group comparison, significant pairwise comparisons: *p* [*PAX3* vs. *SOX10*] = 7.864E-11, *p* [*MITF* vs. *SOX10*] = 0.000001). The ratio of gender showed no significant difference between the different genes. Among those 13 phenotypes, hearing impairment and iris pigmentary abnormality were the most frequent phenotypes in WS patients, which accounted for 84.56% (345/408) and 74.88% (300/402) respectively. Statistical analysis showed that hearing impairment was more frequent in WS probands with *SOX10* variants (*p* = 1.6847E-07 for group comparison, significant pairwise comparisons: *p* [*PAX3* vs. *SOX10*] = 1.0862E-08, *p* [*MITF* vs. *SOX10*] = 0.000007), which is similar to previous reports [7]. In addition, patients with *SOX10* variants were more likely to occur bilateral profound hearing impairment, vestibular deformity, cochlear hypoplasia, and hypoplasia of the semicircular canal (Fig. 6A). We retrieved the expression abundance of *SOX10* in different ear tissues based on the MGI database and found that compared with *PAX3* and *MITF*, *SOX10* was widely and highly expressed in the organ of Corti, semicircular canal, inner ear vestibular component and otocyst (Fig. 6B). Moreover, we found that *SOX10* also had a higher expression level than *PAX3* and *MITF* in cochlear single cells (IHCS, OHCs, DCs) (Fig. 6C). Interestingly, we found that nervous system diseases such as intellectual disability, sensorimotor neuropathy and peripheral demyelinating neuropathy only occurred frequently in patients with *SOX10* variants but were absent in those with *MITF* or *PAX3* variants (Fig. 6A, Additional file 7). RNA-seq data revealed that *SOX10* has a higher expression level than *PAX3* and *MITF* in brain tissues (Fig. 6D). The above results indicated that phenotypic profiles of the *SOX10* gene are closely related to tissue expression patterns.

Pigmentation abnormalities in WS patients mainly included iris pigmentary abnormality (heterochromia iridis, blue irides, and iris hypopigmentation), hypopigmented skin patches, skin freckles, white forelock, and premature graying of hair. Iris pigmentary abnormality occurred frequently in patients with *PAX3* and *SOX10* variants (*p* = 0.004280 for group comparison, significant pairwise comparisons: *p* [*PAX3* vs. *MITF*] = 0.020436, *p* [*SOX10* vs. *MITF*] = 0.002416). Skin freckles and premature graying of hair were frequently observed in patients with *MITF* variants (70.68% and 42.73% respectively). White forelock occurred significantly more often in patients with *PAX3* variants (*p* = 0.004427 for group comparison, significant pairwise comparisons: *p* [*PAX3* vs. *MITF*] = 0.016571, *p* [*PAX3* vs. *SOX10*] = 0.002665). No difference was found in the prevalence of hypopigmented skin patches among different genes. Synophrys and broad nasal root occurred mainly in cases with *PAX3* and *MITF* variants (*p* = 0.004425 and *p* = 0.000635 for group comparison respectively). Telecanthus was frequently present in WS patients with *PAX3* variants (*p* = 2.1124E-32 for group comparison, significant pairwise comparisons: *p* [*PAX3* vs. *MITF*] = 1.0045E-22, *p* [*PAX3* vs. *SOX10*] = 2.5541E-24). It is important to note that aganglionic megacolon occurred frequently in patients with *SOX10* variants (36.00%, 18/50) but was absent in those with MITF or PAX3 variants. Underdeveloped nasal alae was only observed in patients with *PAX3* variants but no difference was found among different genes, mainly because this phenotype was poorly documented in cases with *MITF* and *SOX10* variants. For other rare phenotypes (Additional file 7), the genotype-phenotype associations need to be further confirmed in more WS cases.

Subsequently, we conducted a statistical analysis to investigate whether there is a gender difference in the prevalence of phenotypes. Results revealed that no difference was found in the prevalence of those common phenotypes (hearing impairment, skin freckles, hypopigmented skin patches, premature graying of hair, iris pigmentary abnormality, synophrys) between different genders (Additional file 8). The other phenotypes were not included in the gender analysis due to the small sample size.

## Discussion

In this study, we predicted two possible putative genes (*KIT*, *CHD7*) that may contribute to WS disease development, which provide new clues for the clinical diagnosis of WS. The *KIT* gene encodes a glycosylated transmembrane protein, which is a type III receptor tyrosine kinase. *KIT* was activated through binding of *KITLG*, leading to autophosphorylation at tyrosine residues and downstream signaling cascade responses [41]. Previous studies have reported that several *KIT* variants at the tyrosine kinase domain have been identified in deaf rats and mice [42, 43]. More importantly, Xu et al. have confirmed that the *KIT* variant (NM_001044525.1, c.2418T > A, p.Asp806Glu) destroyed the development of melanocytes in the cochlea, which causes stria vascularis malformation and dysfunction, resulting in degeneration of HCs and finally hearing loss. The pig pedigree with this variant presented congenital bilateral severe sensorineural hearing impairment and hypopigmentation, which is consistent with human WS phenotypes [44]. Collectively, the above evidence further proves that *KIT* is strongly related to the pathogenesis of WS and can be used in the WS-related gene screening clinically. In addition, results from Xu et al. also corroborate the reliability of disease candidate pathogenic genes predicted by integrating the PPI network and the phenotypic-similarity network in our study.

*CHD7*, the gene encoding chromodomain helicase DNA binding protein 7, is an ATP-dependent chromatin remodeler that regulates downstream target gene expression via changing nucleosome accessibility [45]. *CHD7* caused approximately 53% of cases of human CHARGE syndrome, which is a rare syndromic deafness characterized by vision and hearing loss, congenital heart disease, and malformations of craniofacial and other structures [46]. CHARGE syndrome is a condition that partially overlaps with WS, without pigmentary abnormalities. Similarly, a study by Beauregard-Lacroix et al. indicated that *ATP6V1B2* mutation (NM_001693.4; c.1516 C > T; p.Arg506*) could cause DOORS or DDOD syndrome [47]. DDOD syndrome is a rare syndromic deafness that partially overlaps with DOORS syndrome, without seizures and intellectual disability. These results suggest that there could overlap in molecular etiology among different syndromic deafness disorders.

WS is a rare genetic disorder mainly characterized by hearing loss and pigmentary abnormalities. The hearing loss phenotype had been demonstrated in mouse with *CHD7* mutations [48]. Asad et al. also have reported that *CHD7* variants can cause pigmentary abnormalities. The loss of *CHD7* affects the migration of pigment precursors and inhibits the differentiation of pigment lineages in the *CHD7* knockdown zebrafish CHARGE model. Such as, at 5 days post fertilization, zebrafish embryos had iridescent iridophores present in eye and body while iridophores were absent in *CHD7* morphants. Moreover, at 5 days post fertilization, zebrafish embryos had condensed dark melanophores, while *CHD7* morphants had spread out dendritic shaped melanophores with overall less melanin. In addition, *CHD7* morphants had severe downregulation of tyrosinase and dopachrome tautomerase in 24 days post fertilization embryos, which are two enzymes expressed by melanophores that play a crucial role in melanin formation [49]. It is worth noting that CHARGE patients have not been reported to have any defects in pigmentation. However, this phenotype was closely related to WS. In addition, *CHD7* mutations affected neural crest cell development (such as migration, differentiation, and fate choice) and *SOX10* expression from the early stages [49]. Neural crest hypoplasia is the most recognized pathogenic mechanism of WS [50]. *SOX10*, a known pathogenic gene of WS, starts its expression in the late premigratory neural crest and plays key roles in maintaining multipotency of neural crest stem cells, promoting cell survival prior to lineage commitment, and making cell fate decisions for several neural crest derivatives [51]. Therefore, we proposed that *CHD7* could affect the development of neural crest cells by regulating *SOX10* expression and lead to the occurrence of WS. Taken together, we highly suspect that *KIT* and *CHD7* are possible putative WS pathogenic genes.

For the 32 predicted candidate variants (*PAX3*: 20; *MITF*: 7; *SOX10*: 5) from ANNOVAR annotation, we evaluated their pathogenicity based on American College of Medical Genetics and Genomics/Association for Molecular Pathology (ACMG/AMP) guidelines [52]. The 32 predicted candidate variants were classified as “Unknown significance or Pathogenic” according to the ACMG/AMP guidelines (the ACMG classification of these 32 variants was from the DVD database records) [34]. However, the 32 predicted candidate missense variants were predicted as disease-causing by SIFT, Polyphen2, Mutation Taster, CADD, and GERP + + tools. Moreover, multiple alignment with homologous genes from different species with COMBALT showed that the 32 variants are all located in the conserved regions, which suggests that these 32 variants may have a significant impact on protein structure or function.

To further validate the pathogenicity of the 20 candidate variants in *PAX3*, we checked the specificity of their locations. Evidence indicates that 11 mutations (p.Gly34Cys, p.Gly34Arg, p.Gly42Cys, p.Asn47Lys, p.Gly48Ala, p.Val114Met, p.Trp131Ser, p.Asp144Tyr, p.Ser152Asn, p.Arg156Cys, p.Arg156His) are located in the paired domain, which is made up of 128 amino acids in the amino-terminal region of *PAX3*. The paired domain also mediates protein-protein interactions in addition to binding DNA, which impacts the overall function of *PAX3* [53]. Two mutations (p.Arg220Cys, p.Pro244Leu) are located in the homeodomain, which affects the DNA binding ability of the paired domain. In addition, the homeodomain is also capable of interacting with other proteins to regulate *PAX3* activity [53]. The other seven mutations (p.Pro333Leu, p.Tyr366Asn, p.Pro382His, p.Gly417Arg, p.Gln431Glu, p.Tyr458His, p.Gln470Leu) are located in the transactivation domain. The transactivation domain is an S/G/T rich region located in the C terminal tail of the protein, it may directly interact with both the paired and homeodomains and functions to mediate the integrity of *PAX3*-DNA interactions [53]. In all, due to the functional importance of these domain regions, the 20 candidate variants in functional domains are highly likely to cause WS disease.

For the 7 candidate mutations (p.Pro232Arg, p.Arg263Leu, p.Arg263Cys, p.Arg263Gly, p.Arg279Trp, p.Arg279Leu, p.Glu287Lys) identified in *MITF*, we found that these seven mutations all were located in the b-HLH-Zip (basic helix-loop-helix leucine zipper) domain region. The b-HLH-Zip domain regulates DNA binding and dimer formation. In addition, the b-HLH-Zip domain also plays a key role in modulating the transcription factor’s subcellular localization and stability. Pingault et al. reported that single nucleotide variants are not equally scattered along the gene: the majority of them are clustered in the b-HLH-Zip structure [6]. Similarly, we found that almost half of the variations are located in exons 7 and 8 that correspond to the b-HLH-Zip structure (Additional file 5), which further supports the conclusion that the 7 candidate mutations in the b-HLH-Zip structure are most likely to be associated with WS.

*SOX10* possesses four crucial structural domains: the DNA-dependent dimerization domain (Dim), DNA-binding HMG domain, K2 domain and transactivation domain (TA) (Fig. 4). Among the five candidate mutations identified in *SOX10*, two of them (p.Pro245Arg, p.Pro302Leu) are located in the K2 domain, and three mutations (p.Thr437Met, p.Thr461Met, p.Arg465Leu) are located in TA domain. The HMG structure forms an L-shaped module consisting of 3 helices that binds to DNA in the minor groove. This domain is also capable of interacting with partner proteins and regulating intracellular transport. The K2 domain acts as a strong transcription activation domain. This domain regulates protein-protein interactions and co-factor activity [54]. The carboxy terminal TA domain is indeed essential and *SOX10* primarily functions as a transcriptional activator during neural crest development [55]. Therefore, considering the functional importance of these domain regions, we conclude that these five mutations possibly generate a severe effect on people with these variations, and are most likely closely associated with WS. In conclusion, the above evidences all support the pathogenesis of these 32 candidate mutations to WS. Currently, there is no effective therapeutic treatment available for WS. Gene editing has emerged as a promising avenue to treat various disorders, especially genetic diseases in recent years [56–58]. We will construct animal models knocked into these predicted potential pathogenic variants through gene editing technology in follow-up research to study the functions of these predicted variants in WS. Additionally, WS models are constituted using gene editing systems and may develop new methods for the treatment of WS.

Deciphering the genotype and phenotype correlations can promote a better understanding of the disease mechanism and provide help for physicians to better perform clinical diagnosis of rare diseases [59]. Contrary to previously reported results [60], we found that the percentage of premature graying of the hair was a significant difference between patients with *SOX10* and *MITF* variants (*p* = 0.005676). Furthermore, our results showed that no significant difference was observed in the prevalence of hypopigmented skin patches among patients with *PAX3*, *MITF*, and *SOX10* variants. However, Wang et al. study results indicated that hypopigmented skin patches are more often in cases with *PAX3* variants [7]. These differential findings may be due to the small sample size or the limited region and ethnicity of the previous study. In this study, we collected the largest WS cohort with detailed clinical information and these cases from different regions and ethnicities. So, our analysis results could better evaluate the genotype-phenotype correlations.

In this study, we collected a total of 52 phenotypes from 84 published literature, including 24 common phenotypes (Fig. 6A) and 28 rare phenotypes (Additional file 7). Interestingly, we noticed that some phenotypes are exclusively present in patients with specific gene variants. For example, wide nasal bridge and underdeveloped nasal alae were only observed in patients with *PAX3* variants, and cochlear hypoplasia, hypoplasia of the semicircular canal, intellectual disability, and so on only occurred in patients with *SOX10* variants. Therefore, these specific phenotypes can be used for the identification of causative genes.

However, our study also has certain limitations, one of them is that the same phenotypes could be described inconsistently in different cases. Secondly, clinical phenotypes were not fully recorded in many patients. Thirdly, the sample size collected was insufficient. These deficiencies may affect the accuracy of genotype-phenotype association analysis. Thus, detailed and standardized phenotypic records are needed in future studies. In addition, we identified 32 novel potential disease-causing variants associated with WS by ANNOVAR annotation. However, ANNOVAR is only focused on coding region and does not consider the other classes of variants, such as deep intronic, near splice-site, or fusion transcripts. The utility of RNA-seq in diagnosing rare genetic diseases has been demonstrated in several recent studies, which can detect splicing defects and fusion transcripts. Diagnostic rates 35% higher than those previously achievable with DNA-seq alone have been attained [61]. So, the RNA-seq approach can be considered for WS patients with unknown etiology.

## Conclusion

In this study, we predicted two potential pathogenic genes (*KIT*, *CHD7*) and thirty-two potential disease-causing variants, which could potentially contribute to the WS disease. We also provided an overview of the different types of WS and their corresponding genes, and analyzed the correlations between genotypes and phenotypes. Our comprehensive deciphering of the genotype and phenotype association can promote a better understanding of WS in clinical diagnosis and genetic counseling. In addition, our study also provides an alternative way to research new causative factors for rare diseases.

### Electronic supplementary material

Below is the link to the electronic supplementary material.

Supplementary Material 1

Supplementary Material 2

Supplementary Material 3

Supplementary Material 4

Supplementary Material 5

Supplementary Material 6

Supplementary Material 7

Supplementary Material 8

Supplementary Material 9

## Data Availability

Public database resources were listed in Additional file 9. All data generated or analyzed during this study are included in this published article and its Additional files.

## Abbreviations

WS: Waardenburg syndrome
PPI: protein-protein interactions
MGI: Mouse Genome Informatics
IHCs: *inner hair cells*
OHCs: *outer hair cells*
DCs: Deiters’ cells
HPO: Human Phenotype Ontology database
