# Supplementary material for: Deciphering potential causative factors for undiagnosed Waardenburg syndrome through multi-data integration
Source: Orphanet J Rare Dis. 2024 Jun 6;19:226. doi: 10.1186/s13023-024-03220-y (PMC11155130; doi:10.1186/s13023-024-03220-y)
Supplement: Supplementary file 1 — Supplementary Material 1 [file 13023_2024_3220_MOESM1_ESM.pdf]

Additional file 2 The phenotype overview of *KIT*, *CHD7*, *EP300* and *SNAI3* genes with mutations in mice.

	adipose tissue	behavior/neurological	cardiovascular system	cellular	craniofacial	digestive/alimentary system	embryo	endocrine/exocrine glands	growth/size/body	hearing/vestibular/ear	hematopoietic system	homeostasis/metabolism	immune system	integument	limbs/digits/tail	liver/biliary system	mortality/aging	muscle	neoplasm	nervous system	pigmentation	renal/urinary system	reproductive system	respiratory system	skeleton	taste/olfaction	vision/eye
KIT		✓	✓	✓	✓	✓	✓	✓	✓	✓	✓	✓	✓	✓	✓	✓	✓	✓	✓	✓	✓	✓	✓	✓	✓		✓
CHD7	✓	✓	✓	✓	✓	✓	✓	✓	✓	✓	✓	✓	✓		✓		✓	✓		✓			✓	✓	✓		✓
EP300			✓	✓	✓	✓	✓	✓	✓		✓	✓	✓				✓	✓		✓		✓		✓			✓
SNAI3																	✓										
