# Supplementary material for: Deciphering potential causative factors for undiagnosed Waardenburg syndrome through multi-data integration
Source: Orphanet J Rare Dis. 2024 Jun 6;19:226. doi: 10.1186/s13023-024-03220-y (PMC11155130; doi:10.1186/s13023-024-03220-y)
Supplement: Supplementary file 4 — Supplementary Material 4 [file 13023_2024_3220_MOESM4_ESM.pdf]

Additional file 5 Distribution of pathogenic gene variants in gene structure.

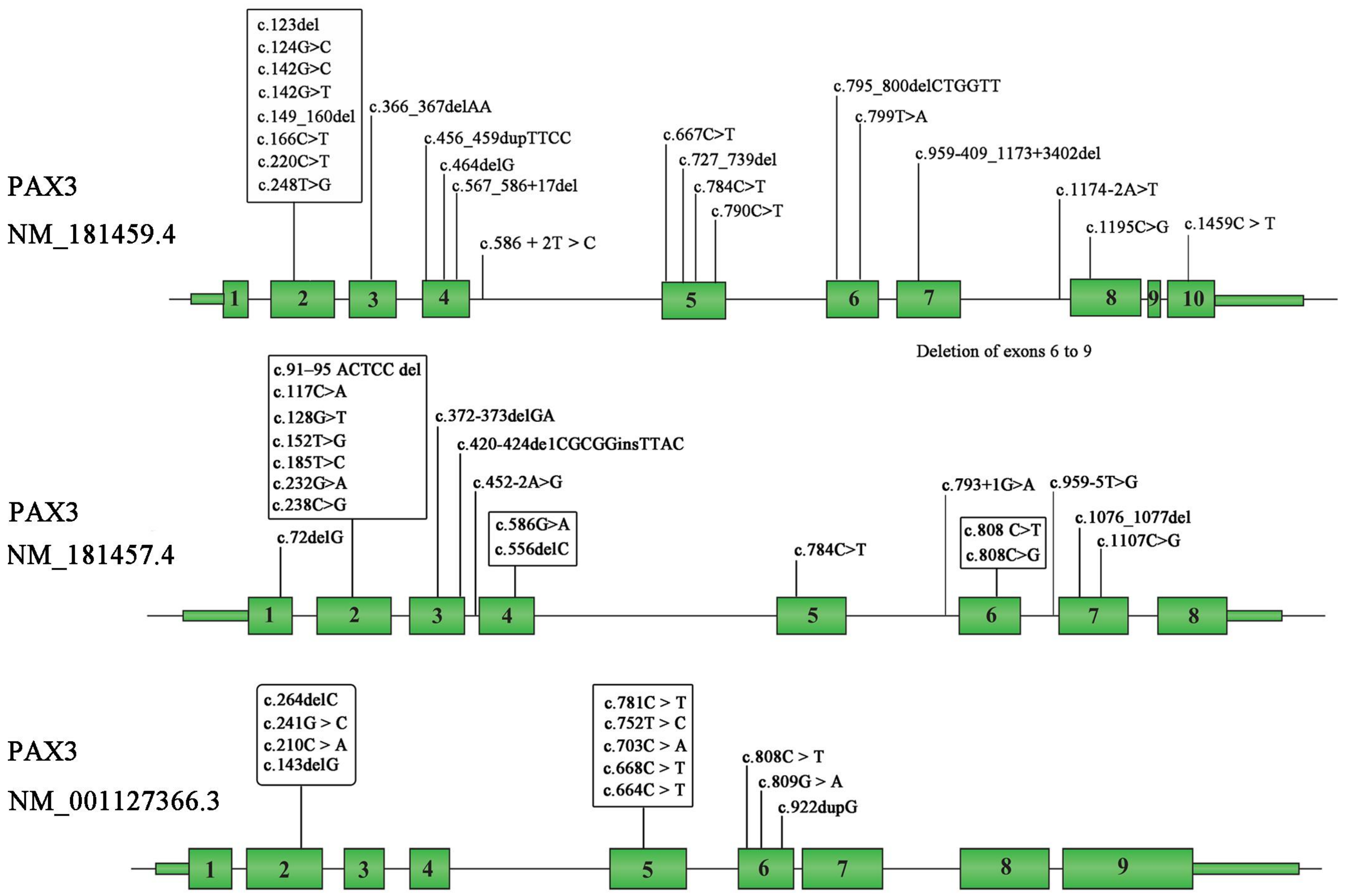

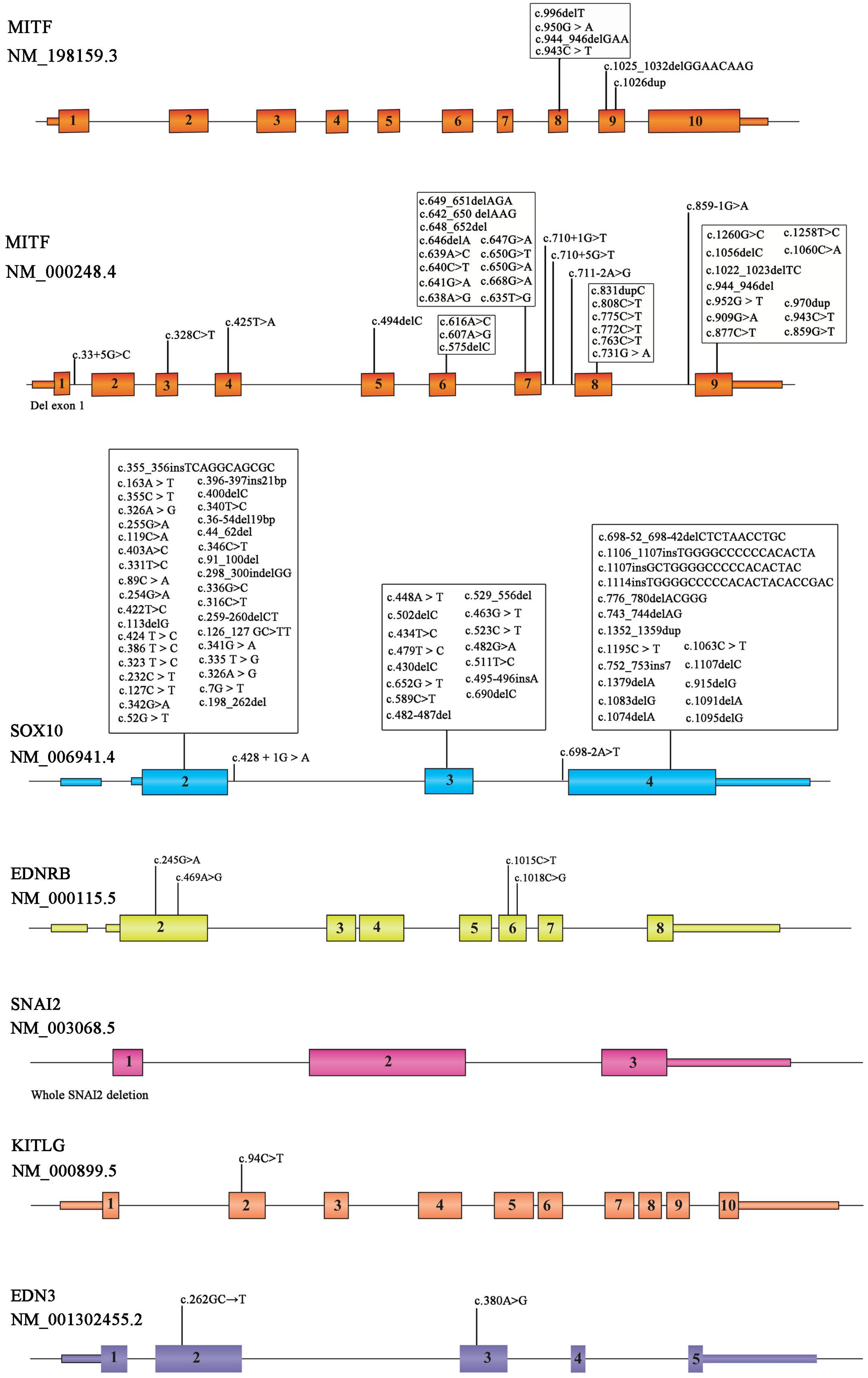
MITF
NM_198159.3
c.996delT
c.950G > A
c.944_946delGAA
c.943C > T
c.1025_1032delGGAACAAG
c.1026dup
1
2
3
4
5
6
7
8
9
10
MITF
NM_000248.4
c.649_651delAGA
c.642_650 delAAG
c.648_652del
c.646delA
c.647G>A
c.639A>C
c.650G>T
c.640C>T
c.650G>A
c.641G>A
c.668G>A
c.638A>G
c.635T>G
c.710+1G>T
c.710+5G>T
c.711-2A>G
c.859-1G>A
c.1260G>C
c.1258T>C
c.1056delC
c.1060C>A
c.1022_1023delTC
c.944_946del
c.952G > T
c.970dup
c.909G>A
c.943C>T
c.877C>T
c.859G>T
c.831dupC
c.808C>T
c.775C>T
c.772C>T
c.763C>T
c.731G > A
c.425T>A
c.494delC
c.616A>C
c.607A>G
c.575delC
c.328C>T
c.33+5G>C
1
2
3
4
5
6
7
8
9
Del exon 1
c.355_356insTCAGGCAGCGC
c.163A > T
c.396-397ins21bp
c.355C > T
c.400delC
c.326A > G
c.340T>C
c.255G>A
c.36-54del19bp
c.119C>A
c.44_62del
c.403A>C
c.346C>T
c.331T>C
c.91_100del
c.89C > A
c.298_300indelGG
c.254G>A
c.336G>C
c.422T>C
c.316C>T
c.113delG
c.259-260delCT
c.424 T > C
c.126_127 GC>TT
c.386 T > C
c.341G > A
c.323 T > C
c.335 T > G
c.232C > T
c.326A > G
c.127C > T
c.7G > T
c.342G>A
c.198_262del
c.52G > T
c.448A > T
c.529_556del
c.502delC
c.463G > T
c.434T>C
c.523C > T
c.479T > C
c.482G>A
c.430delC
c.511T>C
c.652G > T
c.495-496insA
c.589C>T
c.690delC
c.482-487del
c.698-52_698-42delCTCTAACCTGC
c.1106_1107insTGGGGCCCCCCACACTA
c.1107insGCTGGGGCCCCCACACTAC
c.1114insTGGGGCCCCCACACTACACCGAC
c.776_780delACGGG
c.743_744delAG
c.1352_1359dup
c.1195C > T
c.1063C > T
c.752_753ins7
c.1107delC
c.1379delA
c.915delG
c.1083delG
c.1091delA
c.1074delA
c.1095delG
SOX10
NM_006941.4
c.428 + 1G > A
c.698-2A>T
2
3
4
EDNRB
NM_000115.5
c.245G>A
c.469A>G
c.1015C>T
c.1018C>G
2
3
4
5
6
7
8
SNAI2
NM_003068.5
1
2
3
Whole SNAI2 deletion
KITLG
NM_000899.5
c.94C>T
1
2
3
4
5
6
7
8
9
10
EDN3
NM_001302455.2
c.262GC→T
c.380A>G
1
2
3
4
5
