# Supplementary figures and images for: Deciphering potential causative factors for undiagnosed Waardenburg syndrome through multi-data integration
Source: Orphanet J Rare Dis. 2024 Jun 6;19:226. doi: 10.1186/s13023-024-03220-y (PMC11155130; doi:10.1186/s13023-024-03220-y)

**Additional file 7** Rare phenotypes caused by 7 known causative genes in WS.

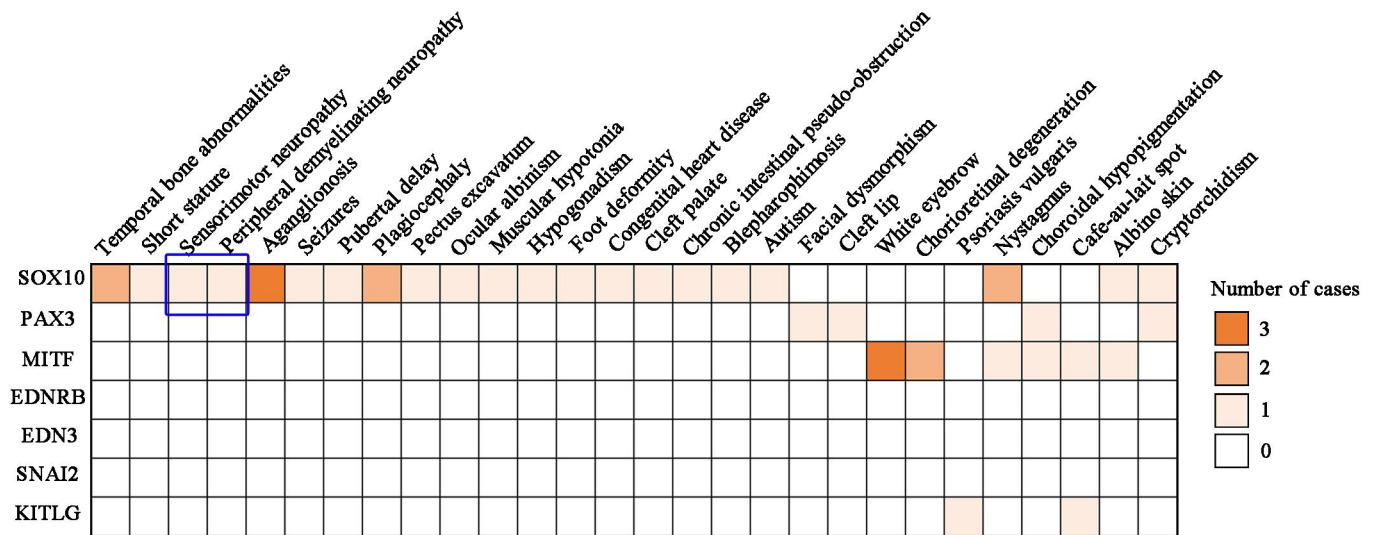

Supplement: Supplementary file 5 — Supplementary Material 5 [file 13023_2024_3220_MOESM5_ESM.pdf]
